# Supplementary material for: Entropy Measures of Human Communication Dynamics
Source: Sci Rep. 2018 Oct 24;8:15697. doi: 10.1038/s41598-018-32571-3 (PMC6200760; doi:10.1038/s41598-018-32571-3)
Supplement: Supplementary file 1 — Supplementary Information [file 41598_2018_32571_MOESM1_ESM.pdf]

Entropy Measures of Human Communication Dynamics - Supplementary Information

Marcin Kulisiewicz[1,*], **Przemysław Kazienko**[1], **Boleslaw K. Szymanski**[2], **and Radosław Michalski**[1]

[1]Wroclaw University of Science and Technology, Department of Computational Intelligence, Wroclaw, 50370, Poland [2]Rensselaer Polytechnic Institute, Department of Computer Science, Troy, NY 12180-3590, US

[*]marcin.kulisiewicz@pwr.edu.pl

In this section, we present additional experimental results. As it was mentioned in main body of this paper, we recognize some potential of entropy measure to distinguished groups in social networks - nodes that share same attribute - based on entropy of their communication. Since it is not main goal of this work we would like to share only proof of concept with preliminary results. We extract communication of several different kind of groups in our datasets. One of the most interesting is looking at groups created based on gender of their members. We look at communication of man and woman separately as well as with each other. We also consider separately communication which take place within a group and between groups. The results presented in Fig.5 show that different groups created based on gender and scope of communication have different values of normalized entropy. Since we have no data to compute entropies of gender based groups in other datasets, it is a preliminary conclusion that gender influences dynamics of interactions. However, entropies of inter and intra group communication are available for majority of examined datasets. Inspecting these entropies, we conclude that intra-group communication tends to have higher entropy than inter-groups communication has. In other words, people seem to know better their group members than non-members. Consequently, they are more selective in their communication with members than non-member. This notion is very interesting and we would like to investigate it in our further works.
We also examined robustness of third-order entropy, which can be difficult to compute correctly when many events occur at the same time. To test robustness of this computation, we add a small random bias to events times, to minimize probability that two events co-occur. We repeat experiment 100 times, each computing third-order entropy. We present results of this experiment as histograms in Fig. 6A for each real dataset we used in previous experiments. We can observe that results concentrate around some mean value with very narrow standard deviation and distribution seems to be normal. Mean value is always higher than original dataset but it is not very distant from original dataset. For the lack of reference point, the question how relevant this distance is remains the subject of the future study.

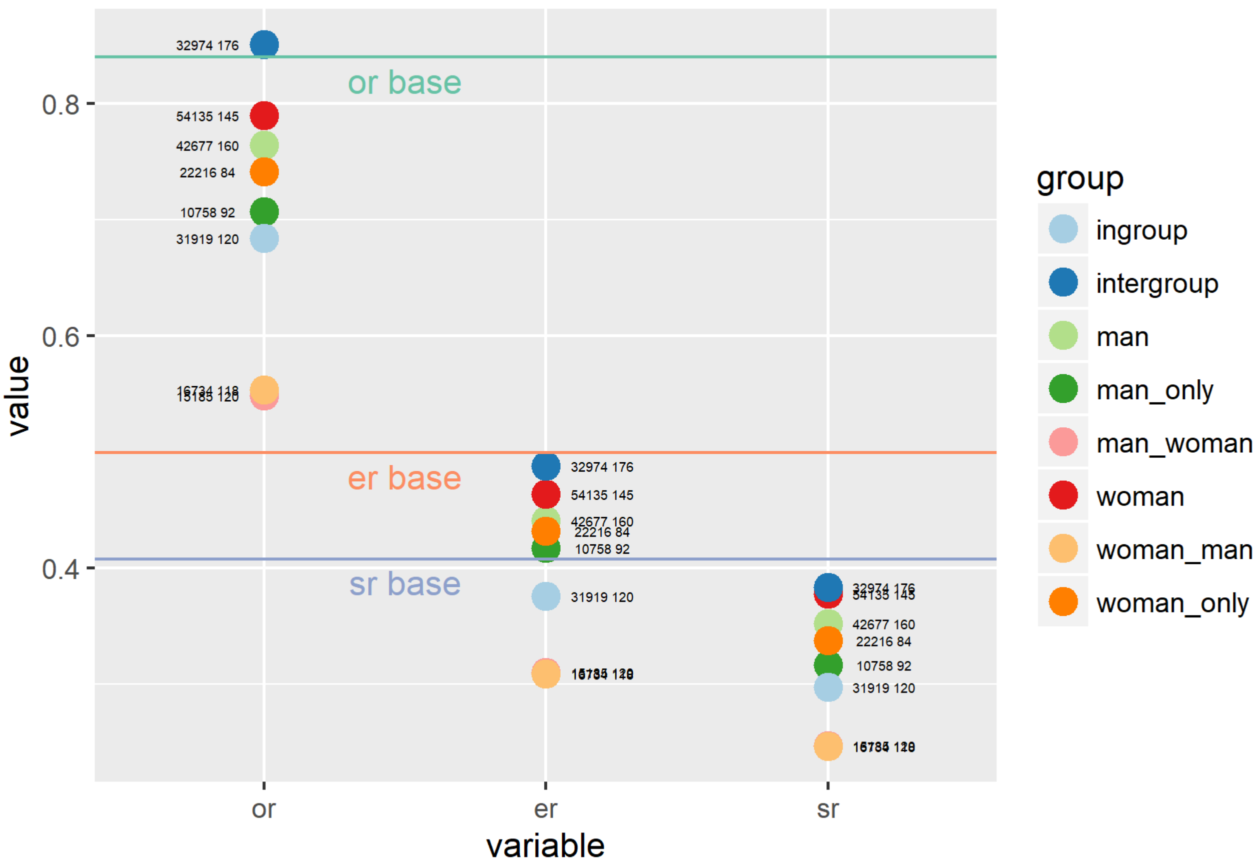

Figure 5. The normalized entropy of groups communication in the Netsense dataset. Groups *man_only* and *woman_only* refers to communication that happens only in those groups while groups *man* and *woman* refers to communication where one of the interlocutors is in the respective group. Groups *ingroup* and *intergroup* refers to communication that respectively occurs between interlocutors that belong to the same and different groups. Numbers next to circles refer to the number of events (contacts) and the number of unique nodes in measured population. Horizontal lines refers to the entropy of entire dataset.

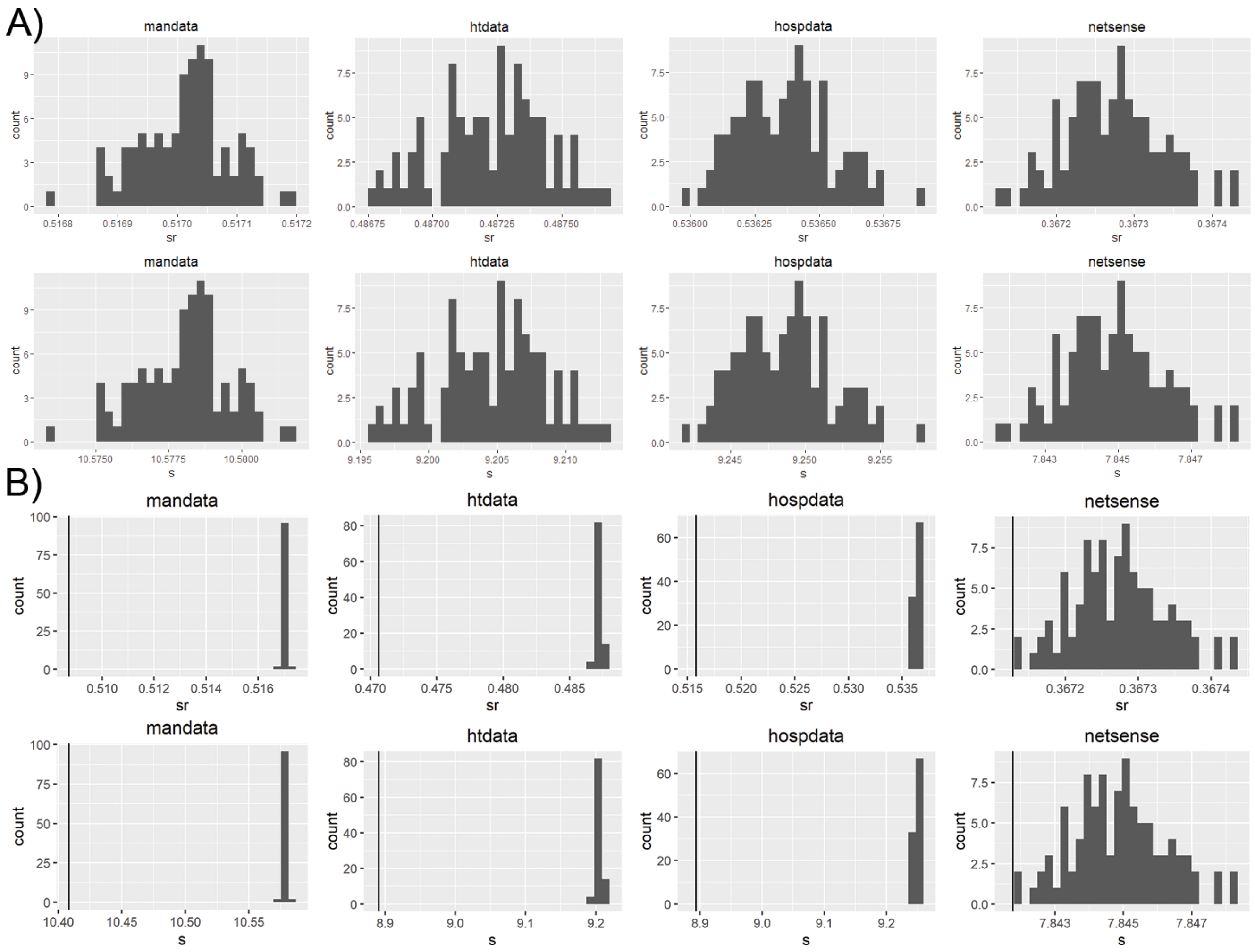

Figure 6. Third-order entropy distribution in biased datasets, results of 100 runs of experiment. A) distribution of third-order entropy in biased dataset B) distribution of third-order entropy in biased dataset with entropy value of unbiased dataset (vertical line).

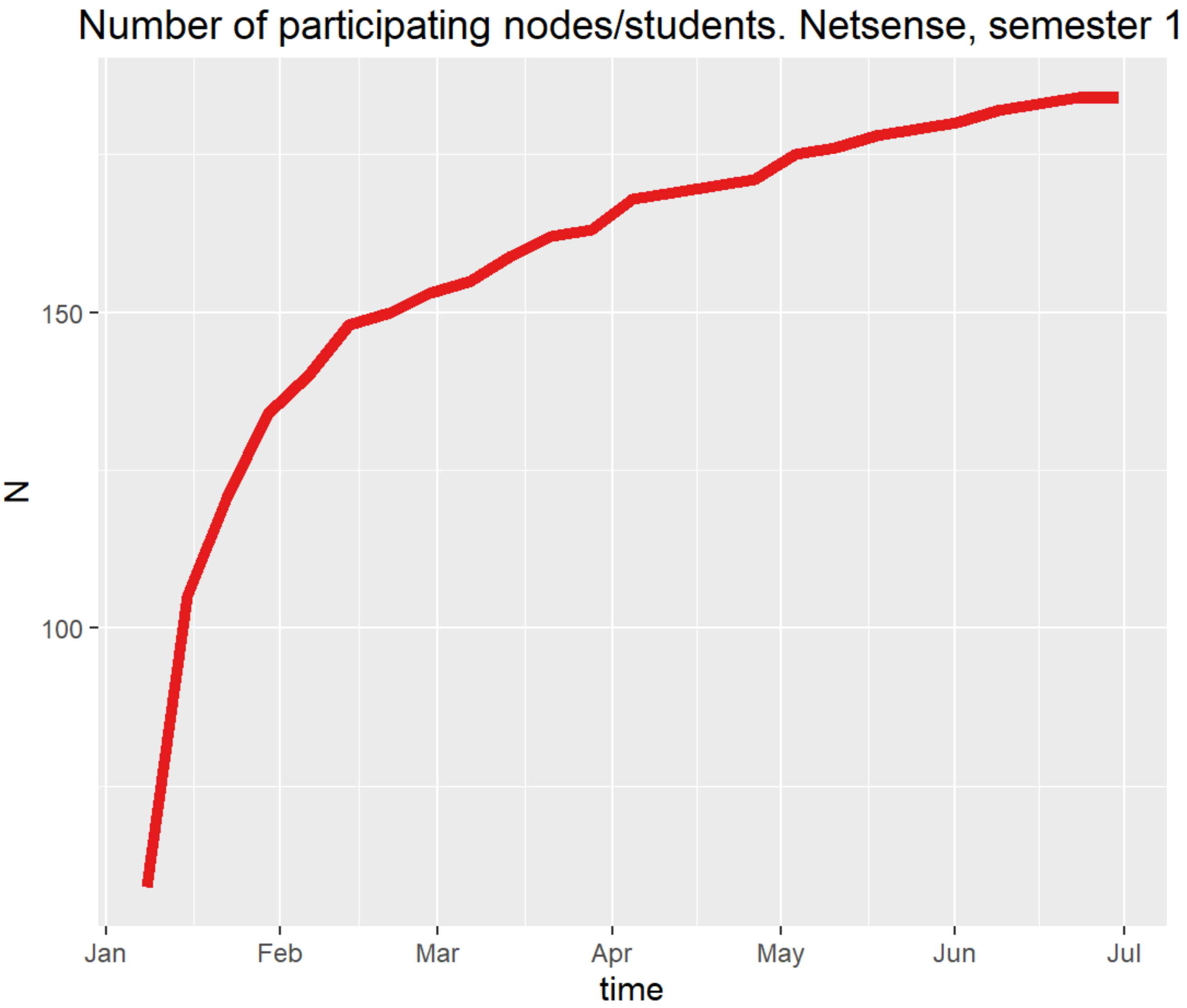

Figure 7. NetSense data: the number N of participating nodes – interacting students – which is non-decreasing over time because of the monotonically increasing set of events. This is also the direct reason for the non-decreasing maximum value of entropy used for normalization.
